# Correlation between cephalic screw positioning of Standard Gamma 3 Nail for intertrochanteric fractures and cut-out incidence

**DOI:** 10.1051/sicotj/2024006

**Published:** 2024-02-26

**Authors:** Alessandro Ortolani, Debora Lana, Antonio Martucci, Francesco Pesce, Stefano Stallone, Lorenzo Milani, Roberto Urso, Giuseppe Melucci, Domenico Tigani

**Affiliations:** 1 Department of Trauma and Orthopaedic Surgery, Maggiore Hospital Largo Nigrisoli 2 40133 Bologna Italy; 2 Division of Renal Medicine, “Ospedale Isola Tiberina - Gemelli Isola” Via di Ponte Quattro capi, 39 00186 Rome Italy

**Keywords:** Proximal femur lateral fractures, Cephalic screw positioning, Cut-out, TAD, CalTAD

## Abstract

*Introduction*: Lateral fractures of proximal femur are the most frequent fractures in elderly people. Internal fixation using medullary nails is the gold standard of treatment (Gamma 3 nail is the most implanted device) due to reduced incidence of complications than other devices. We report our experience in treating this kind of fractures with Gamma 3 nail, between January 2015 and December 2021. *Methods*: We performed a retrospective cohort study of patients treated in our orthopaedic department; level of clinical care is III: 559 patients (431 females and 128 males, with an average age of 85.3 years) with lateral femoral neck fracture. All patients were surgically treated with Gamma 3 standard nail (SGN). We evaluated preliminary X-rays to classify fractures, according to AO-OTA classification and post-operative X-ray to verify cephalic screw position site, according to areas described by Cleveland in 1959: we measured tip-to-apex distance (TAD) and tip-to-apex calcar referred distance (CalTAD). Finally Chang reduction quality criteria (CRQC) for fracture reduction of trochanteric fractures were determined using preoperative or postoperative Antero-Posterior (AP) and lateral radiographs in a Picture Archiving and Communication System (PACS). Incidence of cut-out was evaluated in relation with these parameters. Patients were divided into 2 groups: first group had cephalic screw in optimal positions (5-8-9), the other group had cephalic screw in other positions. *Results*: In 328 patients (58.7%) screw was in positions 5-8-9, in 231 patients (41.2%) screw was in not-optimal position. Median TAD was 19.1 ± 7.0 mm (range = 0.0–50.5); in 463 patients (82.8%) TAD was ≤ 25 mm. Median CalTAD was 21.4 ± 4.7 mm (range = 5.7–39.2); in 105 patients (79.4%) CalTAD was ≤ 25 mm. Cut-out was observed in 8 cases (1.43%). Multivariate analysis showed a significant correlation (p < 0,05) between incidence of cut-out and fracture type 31A2 and with TAD values >25 mm. Cephalic screw position did not influence incidence of cut-out. *Discussion*: In order to obtain fracture healing with a low risk of failure, in particular cut-out, it is necessary to obtain good reduction of fracture and optimal lag screw position in order to achieve a TAD inferior to 25 mm.

## Introduction

Hip fractures represent the most common fractures occurring in elderly patients, affected by osteoporosis, and they are increasing with the rising aging of population in the last few decades [1]. The Gamma Nail has been introduced into clinical use in 1988 for the treatment of trochanteric hip fractures; nowadays Gamma Nail (Stryker Trauma Gmbh Schönkirchen, Germany), which has reached now its third generation of development, is widespread with more than one million patients treated since the introduction of the implant [2].

Recent studies recommend that intramedullary nail should be preferred to extramedullary fixation systems, that allow a good fracture reduction but, at the same time, require the opening of the fracture with higher blood loss, higher risk of infection and pseudoarthrosis. On the other hand, the use of an intramedullary nail is associated with shorter surgical time, less blood loss and early weight bearing, allowing faster healing time. Nowadays, intramedullary nail is the gold standard of treatment for lateral proximal femur fractures [3]. Prospective studies also highlighted its superiority in terms of reducing the rate of intra and post-operative complications (especially cut-out and refracture) compared to previously used systems [2].

This study aims to evaluate the positioning of the lag screw in patients with lateral hip fractures treated using the Standard Gamma 3, and its correlation with cut-out of the lag screw.

## Materials and methods

**Figure 1:**
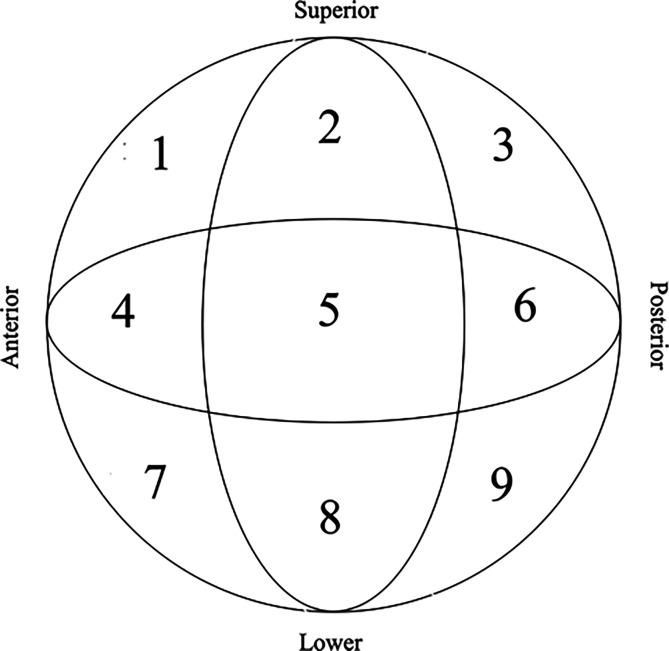
Femoral head position as described by Cleveland.

We performed a retrospective cohort study of 559 patients with lateral proximal femur fracture surgically treated with Gamma 3 standard nail (SGN) at our Orthopaedic and Traumatology Unit in Ospedale Maggiore C.A. Pizzardi of Bologna, during the period between 2015 and 2021. The population of patients consist of 431 females and 128 males, with an average age of 85.3 years (median = 86; range IQR = 81–90, range = 65–103); we recruited patients with a minimum follow-up of 3 months. We evaluated preliminary X-rays to classify fractures according to AO-OTA classification [19] and post-operative X-ray to verify cephalic screw position site and quality of reduction. For the assessment of the cephalic screw position the femoral head was divided into three portions both on the AP view (superior, central, inferior) and on lateral views (anterior, central, posterior) following classification described by Cleveland et al. [3] (Figure 1). Reduction quality was assessed on 3 grades, following the Chang reduction quality criteria (CRQC) [4]. Two criteria were assessed on AP and lateral views: alignment (normal or slight valgus neck-shaft angle, no more than 10°, on AP view and angulation <20° on lateral view), and displacement (<4 mm between each fragment on each view). For each criterion a positive score of 1 or a negative score of 0 is assigned. Reduction was considered excellent in case of a score of 4, acceptable if the score was 2 or 3 and poor for a score of 1 or 0. Based on position of the lag screw and according to evidences suggested by literature [5–10], we decided to separate patients in two groups: in the first group, the lag screw was placed in position 5, 8 and 9 (considered as optimal positions) whereas in the second group the lag screw was placed in other positions (1, 2, 3, 4, 6, 7). Taking into account the importance of TAD and CalTAD as suggested by different authors in literature [11], on immediate postoperative radiographs TAD and CalTAD were measured through the help of an in-house picture archiving and communication system (PACS) tool [11]. Furthermore we took into consideration the possible correlation between the quality of the reduction and the incidence of cu out. Fracture callus healing and appearance of cut-out were evaluated on follow-up radiographs.

The SGN has been positioned according to the standard technique [12], reaming the intra medullary canal 2 mm more than the nail diameter (till using a 13 mm reamer) and reaming 15 mm proximally; cephalic screw was positioned after insertion of the specific guide wire; distal screw was predominantly placed in a static configuration (534 cases of 559, 95.5%). All patients were subjected to early progressive weight bearing within the first week, depending on the general subjective clinical conditions.

Aim of the present study was to evaluate the association between cut-out finding (yes/no) and age (years), gender (males vs. females), type of fracture, screw position (5-8-9 vs. others), TAD (>25 mm vs. ≤25 mm) and CalTAD (>25 mm vs. ≤25 mm).

### Statistical analysis

The study model is retrospective cohort.

The collected data were entered into an Office Excel database and analyzed using Stata MP16 software.

Continuous variables were expressed as mean standard deviation, median, interquartile range (IQR) and range, categorical variables as proportions, with the indication of the 95% confidence interval (95%CI) where deemed appropriate. Fisher’s exact test was used to compare categorical variables between groups.

To calculate the prevalence of cut-out in the sample, the cut-out event was used as the numerator and the number of patients as the denominator; the result was subsequently multiplied by 1000.

To evaluate the association between cut-out findings (YES/NO) and age (years), gender (male vs. female), fracture type, screw position (5-8-9 vs. other), Tip-Apex (>25 mm vs. ≤25 mm) and CalTAD (>25 mm vs. ≤25 mm) a multivariable logistic regression model was used; the adjusted Odds Ratio (aOR) were calculated, with the indication of the 95% CI and the p-value. The Hosmer-Lemeshow test was used to evaluate the goodness-of-fit of the model.

Progression-free survival (PFS) was assessed as the time between the date of surgery and the cut-out date or the date of surgery and the date of the last available follow-up.

The Kaplan–Meier curve was used to evaluate PFS, and the log-rank test was used to compare it between groups. The cut-out incidence rate ×1000 person-months was estimated, with the indication of 95% CI. Subsequently, the Incidence Rate Ratio (IRR) values between groups under analysis were calculated, with relative 95%CI.

To evaluate the risk predictors of PFS, a multivariable Cox semiparametric regression model was used, using age (years), gender (male vs. female), fracture type, screw position (5-8-9 vs. other), Tip-Apex (>25 mm vs. ≤25 mm) and CalTAD (>25 mm vs. ≤25 mm); the adjusted Hazard Ratio (aHR) were calculated, with the indication of the 95% CI and the p-value. The Schoenfeld residual test was used to evaluate the proportionality assumption of the model.

For all tests, a 2-sided *p*-value > 0.05 was considered significant.

Chi-square analysis searching for correlations between reduction-quality and cut-out was performed using IBM Corp. Released 2015. IBM SPSS Statistics for Windows, Version 23.0. Armonk, NY: IBM Corp; a *p*-value < 0.05 was defined as statistically significant.

## Results

**Figure 2:**
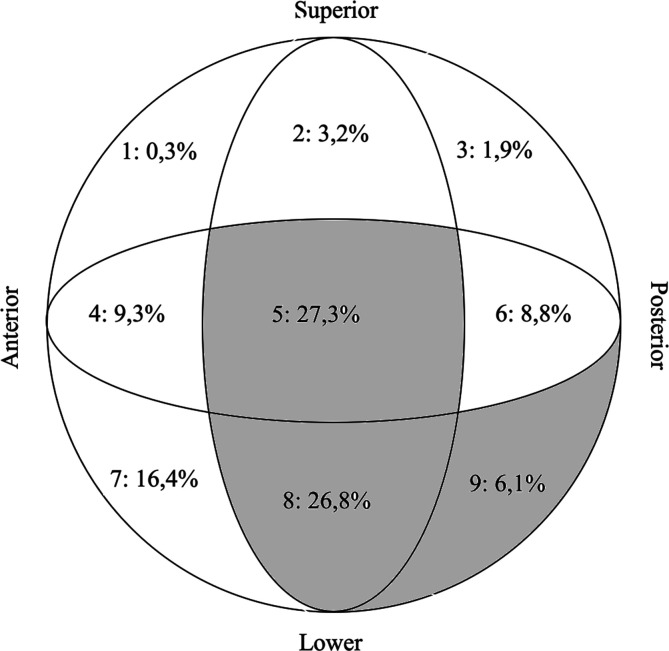
Distribution (%) of screw insertion position.

According to AO-OTA classification [13] 271 cases belonged to 31-A1 fractures, 246 cases to 31-A2 and the remaining 42 cases to 31-A3. The general analysis of the cephalic screw positioning revealed that regions 5 and 8 were the most frequent, while the superior placement of the lag screw on the femoral head (positions 1, 2 and 3) was less frequently represented; whole distribution is detailed in Figure 2, below depicted.

We observed an amount of eight cases of cut-out in our series with a predominance of 1.43 cases every 100 patients.

All cut-out occurred within first six weeks after surgery (range from 4 to 6). Six patients (66.67%) didn’t receive any surgical treatment after this complication due to their advanced age, major medical comorbidities and low functional demands. One patient underwent total hip replacement. In one case we removed only the cephalic screw that was completely ejected into surrounding muscles.

We observed that the lag screws migrated anteriorly-superiorly compared to its intraoperative position in four cases. There weren’t cases of lag screws migrated posteriorly. Central cut-out (along the lag screw axis) occurred in four cases.

If we consider the cephalic screw position within the index procedure, we noticed that in 5 cases of cut-out the screw has been implanted in a position considered as optimal: position 5 in three cases, position 8 in one case, position 9 in one case. Only in three cases the cut out occurred when the screw was implanted in less favourable position (two cases in position 7, one case in position 6). Otherwise, if we consider the kind of fracture, we observed one case of cut-out in A1 fractures, eight cases in A2 fractures and no cases in A3 fractures.

Between the 8 patients who reported cut-out, 6 had excellent reduction quality according to Chang et al. criteria [4] characterized by five positive medial cortex support and two neutral position. In the remaining two patients the quality of the reduction was considered acceptable; one of them presented a negative medial cortex support. In the entire cohort the quality of reduction was excellent in 67% of the cases, with a predominance on positive medial cortex support among neutral position 75% vs. 25%). Acceptable reduction quality has been detected in 29% of cases, most of them with positive medial cortex support which reaches 70% of the total. The remaining 30% was distributed in 21% of neutral position and 9% of negative cortex support. Finally 4% of cases had a poor reduction quality; the negative cortical support reached in this group 60% of cases

Cut-out rate was 6.9% in good reduction, 9% in acceptable reduction and 0% in poor reduction. There were no significant differences in cut-out according to Quality reduction, according to Chi-square analysis.

The multivariate analysis showed a significant statistical correlation between incidence of cut-out and A2 fracture (aOR = 9.1; 95%CI = 1.1–76.1; *p* < 0.05); also TAD values > or = 25 mm is significantly correlated with incidence of cut-out (aOR = 9.1; 95%CI = 1.1–76.1; *p* < 0.05). From our analysis appears that cephalic screw position is not correlated with incidence of cut-out: there is no difference between two groups previously described (position 5-8-9 vs. other positions, neither we observed any correlation with other factors (Table 1).

**Table 1:** Multivariate analysis.

Factors	aOR	95%CI	*p*-value
Age (years)	0.98	0.91–1.05	0.527
Gender (male vs. female)	0.5	0.1–3.0	0.409
Fracture type			
2 vs. 1	9.1	1.1–76.1	0.042
3 vs. 1	1	–	–
Screw position (5-8-9 vs. other)	1.2	0.3–5.5	0.788
Tip-Apex Distance (>25 mm vs. ≤25 mm)	5.5	1.1–28.5	0.043
CalTAD (>25 mm vs. ≤25 mm)	1.4	0.3–7.7	0.696

## Discussion

The prevalence of cut-out complication in our series was of 1.43% (eight cases) in line with other latest large series [2, 14]. In the past, cut-out incidence in different compression hip screw and intramedullary nail reached values even up to 20% [15, 16]. Cut-out is considered a multifactorial event that can be affected by different variables such as age, bone quality, fracture pattern, quality of fracture reduction, lag screw positioning and length, implant design [11, 14]; there is no clear consensus either on interrelationships of all these factors or on relative importance of each one. Considering all these factors, it is easy to understand how surgeons should avoid any technical mistake during positioning of intramedullary device. The optimal position of the lag screw has been widely discussed in the literature, particularly the aspect of central [17, 18] or inferior [2, 8, 14, 19, 20] placement of the screw in the femoral head as seen on the A-P view. In literature there is wide consensus about central position of the screw on lateral view [2, 14, 17–20], however it is still not possible to define the ideal position on the AP view with two main ideas: the deep central placement [17, 18] and the inferior placement [2, 8, 14, 19, 20]. Kuzyk et al. [8] describe in a biomechanical study that cephalic screw inferiorly placed gives the highest axial and torsional stiffness; similar results were obtained by Goffin et al. [9] in a finite elements study in which they suggested that inferior middle and inferior posterior placement should be preferred. Even the technique of Gamma 3 Nail [12] detailed by Taglang suggests the AP placement in the inferior part of the neck. Some authors [8, 9] suggested that the inferior placement of the lag screw causes a TAD increase compared with the central placement; this increase is due to the fact that lag screw is not directed towards the apex of the femoral head [20]. According to these results we decided to compare the case where lag screw was implanted in position 5, 8 and 9, as described by Cleveland et al. [3] and considered optimally placed, with all other positions.

In our study the incidence of cut-out (eight cases on 559 patients with an age superior to 65 years old) appears to be unrelated to cephalic screw position, in particular we did not find a real protective effect in placing the screw in position 5, 8 and 9. In fact we observed 5 cases of cut out on 328 patients (1.53%), while in all the other positions we detected only 3 cut-out on 231 patients (1.30%), taking into account that these data were no statistically significant (Table 1). Results of our study on lag screw positioning do not confirm as a relevant factor the inferior placement on AP view to reduce incidence of cut-out in treatment of intertrochanteric fractures with intramedullary nail. Probably other factors could be more relevant in this regard, such as the length of the screw and in particular a TAD value <25 mm [8–10]. In our research we observed a TAD <25 mm in about 82.2% of patients; Considering patients with a TAD value >25 mm (96/559), we observed a more relevant cut-out rate (4 patient on 96; 4.16%); on patient’s group with a TAD value <25 mm (463/559) 4 cases developed cut-out on 463 (0.86%). Regarding CalTAD, instead, in our research we observed a CalTAD < 25 mm in about 79.4%. Considering patients with a CalTAD value >25 mm (105/559), we observed cut-out in 3 patients (2.86%); on patient’s group with a TAD value <25 mm (444/559) 5 cases developed cut-out on 105 (1.13%).

According to our results we can conclude that TAD still represents a main predictor of cut-out as shown in literature by many authors [8, 11, 21], but it is not the only one to take into account: poor bone quality in the surrounding cephalic screw or a comminuted intertrochanteric fracture, or non-anatomical reduction of fracture can also lead to cut-out [1].

According to some authors a poor quality of reduction increases cut-out risk [2, 4, 21]. In the present series, only two cases of cut-out occurred in patients with acceptable reduction quality and in no case with poor reduction. The others six cut-out involved patients with excellent reduction.

Considering that cut-out could be due to other factors such as an unstable and complex fracture pattern, we observed almost all cases of cut-out occurring in A2 type fracture: we underline a statistical difference with stable fractures (A1 type). To our knowledge, this correlation between increase of cut-out and A2 type fractures is not clearly described in literature, but if we consider that A2 fractures are unstable, it could be supposed that cut-out is due to increased load on poor quality bone around the lag screw; this increased load could lead to collapsing of the neck-shaft angle into varus and to the extrusion of the screw from the femoral head (i.e. cut-out).

Our research has some limitations. The retrospective nature of the study represents the principal one, due to the non-standardized methods of data collection or inconsistent quality of radiographs. Unfortunately we also have lack of data on patients’ bone quality and that could be considered an important bias to our study; further investigations on correlation of cut-out and poor bone quality should be addressed in future studies. Last potential limitation of our research concerns the cephalic screw position, that is estimated on two projections radiograph and not on a CT scans; however, the latter ones are not routinely necessary after surgery. Nevertheless, this is one of the largest studies from a single Institution with a single type implant.

About raising age population and limited amount of healthcare resources, it is more and more important to avoid any complication when treating hip fractures in elderly people. Elderly patients with hip fractures are extremely fragile and most of them cannot endure a second surgery, which is usually more invasive with higher perioperative life-risk. [22, 23] Without considering a second surgery, mortality rate in elderly patients treated for hip fractures is quite high: this rate is estimated about 5–10% within the first month after surgery, and around 20–30% during the first year. Males have higher mortality risk, that have been shown to persist for up to 10 years [22, 23]. In case of a second surgery, mortality rate is reasonably higher, but also outcome appears to be worse: patients will need to restart another rehabilitation program for an extended period of time and deal with problems associated with a prolonged separation from home environment [22].

In conclusion, in order to reduce the risk of cut-out after intramedullary nail of proximal femur fracture and consequently reduce risk of re-intervention on elderly patients, surgeons should obtain a good and outstanding anatomical reduction of fracture and choose the correct lag screw length in order to reduce TAD value as much as possible. Despite the results achieved, our suggestion is to continue placing cephalic screw into the three zones described as more stable (central-central, inferior-central, inferior-posterior), nevertheless we underline that is more important to achieve good anatomical reduction of fracture and obtain a TAD value < 25 mm or less, especially if we are treating a 31-A2 fracture. However, further investigations should be carried on to understand the correct relevance of each factor in incidence of cut-out.

## Data Availability

Derived data supporting the findings of this study are available from the corresponding author on request.
